# Clinical profiles and hospitalization patterns among abused children referred by Child Guidance Centers in Japan

**DOI:** 10.1007/s44192-025-00360-w

**Published:** 2025-12-18

**Authors:** Koichi Furuhashi

**Affiliations:** 1 Department of Psychiatry, School of Medicine, Fujita Health University, 1-98 Dengakugakubo, Kutsukake-cho, Toyoake, Aichi 470-1192 Japan; 2 Department of Child and Adolescent Psychiatry, NHO Higashiowari National Hospital, Nagoya, Japan

**Keywords:** Gender, Neglect, Neurodevelopmental disorder, Physical abuse, Psychological abuse, Sexual abuse

## Abstract

**Background:**

Child Guidance Centers (CGCs) play a central role in child protection in Japan, yet evidence on abused children referred to psychiatric services remains limited.

**Methods:**

We retrospectively reviewed 84 children referred by CGCs (2019–2023) to a child and adolescent psychiatry department. Demographics, types of abuse, neurodevelopmental disorders (NDs), prior psychiatric care, hospitalization, and experiences of sexual violence by non-caregivers were extracted from medical records and CGC referral forms. Fisher’s exact test, Pearson’s chi-square test, and the Wilcoxon rank-sum test were used, and odds ratios (ORs) with 95% confidence intervals (CIs) were calculated.

**Results:**

Psychological abuse was most prevalent (80%), followed by physical (70%), neglect (35%), and sexual abuse (33%). Girls had higher odds of sexual abuse (OR 6.16, 95% CI 1.32–28.8) and were more frequently hospitalized. Children with NDs were less likely to have sexual abuse recorded (OR 0.29, 95% CI 0.10–0.83). Hospitalization was associated with experiencing a greater number of abuse types and with sexual abuse (OR 4.23, 95% CI 1.49–12.00), particularly when perpetrated by non-caregivers (OR 4.53, 95% CI 1.51–13.60).

**Conclusions:**

Among CGC-referred children receiving psychiatric care, notable patterns included gender differences in abuse profiles, a high cumulative burden of maltreatment, and a lower recorded prevalence of sexual abuse among those with NDs. These findings highlight the need for careful clinical assessment and coordinated, ND-sensitive approaches that reduce under-recognition. Although derived from a CGC-based referral system, the results may inform efforts to integrate child protection and child and adolescent mental health services internationally.

**Supplementary Information:**

The online version contains supplementary material available at 10.1007/s44192-025-00360-w.

## Background

Child abuse is a pervasive public health concern worldwide, with the World Health Organization (WHO) estimating that nearly one billion children experience some form of violence each year [1]. Beyond immediate physical harm, childhood maltreatment is associated with long-term emotional, psychological, and social consequences.

A robust body of empirical research has demonstrated that being maltreated in childhood substantially increases the risk of developing psychiatric disorders, including depression, anxiety, and post-traumatic stress disorder [2, 3]. These associations have been reinforced in recent systematic reviews and meta-analyses, particularly highlighting the strong link between childhood sexual abuse (CSA) and post-traumatic stress symptoms as well as other mental health difficulties in children and adolescents [4, 5]. Among the various forms of maltreatment, CSA has been repeatedly described as one of the most clinically impactful experiences, given its strong association with acute psychological distress and trauma-related symptoms. Experiencing multiple types of maltreatment has also been associated with more complex and severe psychiatric outcomes in youth [6, 7].

Within the framework of developmental psychopathology, the concept of latent vulnerability suggests that early adversity alters emotional and cognitive systems, increasing susceptibility to later mental health difficulties [8]. The Adverse Childhood Experiences Study similarly highlighted the cumulative impact of early trauma on long-term physical and psychological health outcomes [9, 10]. Together, these findings underscore the need for timely mental health interventions for children who have experienced abuse.

In Japan, the issue of child abuse has become increasingly pressing. According to the Ministry of Health, Labour and Welfare (MHLW) [11], Child Guidance Centers (CGCs) nationwide handled 225,509 cases of suspected child abuse in the 2023 fiscal year, marking a record high. CGCs are the primary public agencies responsible for managing cases of abuse, including decisions regarding temporary custody, protection, and referrals for specialized services. While psychiatric services are involved in some of these processes, the extent and consistency of such involvement are known to vary across regions, reflecting differences in local resources and coordination practices.

Homma [12] emphasized the need for full-time child psychiatrists within CGCs, noting that many centers continue to rely on part-time or consulting specialists. Although psychiatric care for abused children is often provided through referrals to medical institutions, these practices are typically shaped by local arrangements rather than a standardized national framework. A report by the Japanese Society for Child and Adolescent Psychiatry [13] similarly described current CGC practices in responding to child sexual abuse and collaborating with medical institutions, highlighting both increasing demand for psychiatric evaluations and the limited number of available specialists. While regional disparities have not been systematically evaluated, structural factors such as workforce shortages and variation in interagency coordination likely contribute to differences in service delivery across areas. These contextual features indicate that access to psychiatric expertise within the CGC system can vary considerably by region, forming an important backdrop for understanding the referral pathways examined in the present study.

International literature on child protection has highlighted that effective interagency collaboration depends on factors such as clearly defined roles, shared protocols, and consistent communication [14]. At the same time, barriers including organizational fragmentation and limited information sharing have also been noted [14, 15]. These findings indicate that coordination among agencies can be challenging even in well-resourced systems.

Previous studies have examined interagency collaboration in various contexts, including coordination for families affected by parental mental illness or substance misuse [16, 17], and have also noted challenges in integrating psychiatric services for abused children within multidisciplinary teams. For example, Subramaniyan et al. [18] described barriers to psychiatric involvement in a hospital-based collaborative response unit, illustrating how structural and communication-related limitations can hinder effective coordination. These findings suggest that successful collaboration requires not only structural and policy alignment but also sustained interprofessional communication and coordinated decision-making—challenges that are particularly relevant when psychiatric expertise is needed in child protection settings.

Although systems through which CGCs can refer children to psychiatric institutions exist in Japan, their implementation varies across regions and is shaped largely by local coordination practices rather than by a standardized national framework. Such referrals typically occur within cooperative interagency arrangements rather than through legally mandated procedures. As a result, access to psychiatric services may differ by region, reflecting variations in available resources and the strength of local collaboration—a contextual factor that informs how referral pathways function in the present study.

Despite the presence of referral pathways, little is known about the clinical characteristics of children who are actually referred from CGCs to psychiatric settings. Clarifying these profiles is essential for understanding how mental health concerns are recognized and managed in practice. Against this background, the present study retrospectively examines children referred from a single CGC to a child and adolescent psychiatry department, with particular attention to patterns of maltreatment, the role of neurodevelopmental disorders, and factors associated with psychiatric hospitalization.

## Methods

This study is a retrospective observational analysis and did not involve a clinical trial.

### Study objectives

In this study, we addressed these gaps by analyzing the clinical characteristics of children referred by CGCs to the Department of Child Psychiatry at our hospital. Specifically, we examined the types of abuse and their prevalences, the role of neurodevelopmental disorders (NDs), and factors associated with psychiatric hospitalization. By providing insights into gender differences, patterns of abuse, and clinical characteristics at the time of referral, this study sought to contribute to the global discourse on child maltreatment and to inform the development of culturally responsive mental health services in Japan. Although the findings are situated within Japan’s CGC-based referral system, they may offer useful insights for enhancing interagency collaboration and mental health service delivery in other countries with similar multidisciplinary structures.

### Study design and setting

In this retrospective observational study, we analyzed the clinical data of children referred by CGCs to the Department of Child Psychiatry at our hospital between April 2019 and March 2023.

The reporting of this study adheres to STROBE (Strengthening the Reporting of Observational Studies in Epidemiology) guidelines, with key elements addressed where applicable.

### Participants

A total of 91 children were referred by CGCs during the study period. Of these, seven were excluded because they were not officially recognized by the referring CGCs as having been abused. The final sample consisted of 84 children with confirmed histories of abuse. The flow of children from CGC referral to psychiatric assessment and inclusion for analysis is illustrated in Fig. S1. The inclusion criteria were referral by a CGC and official confirmation of abuse by the referring CGC. In practice, CGCs confirm abuse through multidisciplinary case conferences, review of interviews with the child and caregivers, and assessment of collateral information (e.g., school or community reports), and this confirmation is documented in the official CGC referral forms. Children without such confirmation were excluded from analysis. This retrospective observational study did not involve prior sample size or power calculations, as all eligible cases within the study period were included.

Children were referred to our department when CGC caseworkers, psychologists, and consulting child psychiatrists jointly determined that specialized psychiatric assessment or treatment was required. Psychiatric referral by CGCs was typically initiated when significant emotional or behavioral dysregulation, safety concerns, or suspected trauma-related symptoms were identified. Decisions regarding hospitalization at our department were based on clinical severity, acute risk (e.g., suicidality or inability to maintain safety), and the need for structured assessment or stabilization rather than on resource availability alone. At the first visit to our hospital, board-certified child psychiatrists evaluated each child and decided whether outpatient care or psychiatric hospitalization was indicated.

### Data collection

Data were extracted from medical records and referral forms provided by the CGCs. Data for the following variables were collected: demographic characteristics of age and gender; types of abuse—including physical, psychological, and sexual abuse as well as neglect—as defined by the national guidelines of the MHLW [19]; clinical characteristics of presence of NDs (e.g., attention deficit/hyperactivity disorder, autism spectrum disorder), history of psychiatric outpatient visits, hospitalization for psychiatric treatment, and experience of sexual violence by non-caregivers. Sexual violence by non-caregivers was defined as sexual acts perpetrated by individuals other than caregivers, such as acquaintances, teachers, or strangers.

In Japan, the statutory definition of “sexual abuse” used by CGCs refers exclusively to sexual victimization perpetrated by caregivers (e.g., parents or guardians). This definition is narrower than the international usage of child sexual abuse (CSA), which typically encompasses all forms of sexual victimization of minors regardless of the perpetrator. Therefore, in this study we classified caregiver-perpetrated sexual victimization as “sexual abuse,” while sexual victimization by non-caregivers (e.g., siblings, peers, teachers, acquaintances, or strangers) was recorded separately as “sexual violence by non-caregivers.”

Neurodevelopmental and psychiatric diagnoses were primarily made by board-certified child psychiatrists at our hospital using the *Diagnostic and Statistical Manual of Mental Disorders*,* Fifth Edition* (DSM-5) criteria. In some cases, diagnoses documented in CGC referral forms were made by affiliated specialists, including child psychiatrists working within CGCs.

### Statistical analyses

Descriptive statistics were used to summarize demographic and clinical characteristics. Categorical data were analyzed using Fisher’s exact test. The Wilcoxon rank-sum test was used for comparisons involving continuous variables (e.g., age). Pearson’s chi-square test was applied where appropriate; Fisher’s exact test was used when expected cell counts were fewer than five. All statistical analyses were performed using EZR version 1.64, a user-friendly statistical software based on R and R Commander, developed specifically for medical statistics [20].

A *p* value < 0.05 was considered statistically significant. Data are presented as number and percentage for categorical variables and as median and interquartile range (IQR) for continuous variables. Cases with missing data were excluded pairwise from relevant analyses. Because this study was exploratory in nature, no adjustments were made for multiple comparisons, and the results should therefore be interpreted with caution.

To explore clinically significant associations underlying referral and hospitalization patterns among abused children, we conducted four comparisons based on predefined groups: gender (girls vs. boys) and types of abuse (Fisher’s exact test); presence or absence of ND and types of abuse (Fisher’s exact test); hospitalization status (hospitalized vs. not hospitalized) and clinical variables (Wilcoxon rank-sum and Fisher’s exact or chi-square tests); presence or absence of sexual abuse and associated factors (Wilcoxon rank-sum and Fisher’s exact or chi-square tests).

### Use of large language models in the writing process

During the preparation of this work, the author used ChatGPT (OpenAI) to refine English expressions, organize sentence structure, and respond to copyediting comments. After using this tool/service, the author reviewed and edited the content as needed and takes full responsibility for the content of the published article.

## Results

### Participant characteristics

Eighty-four children were referred by CGCs to our Department of Child Psychiatry during the study period. The median age was 14 (IQR 12–15) years, and 64 (76%) were girls (Table 1). Thirty-four children (41%) had a history of psychiatric outpatient visits, and 33 (39%) were diagnosed with NDs. Sexual violence by non-caregivers was reported in 18 cases (21%).

**Table 1:** Characteristics of the abused children referred by child guidance centers (*n* = 84)

Variable	
Age (years)	14 [12–15]
Gender: girls	64 (76)
History of psychiatric visits	34 (41)
Neurodevelopmental disorders	33 (39)
Sexual violence by non-caregivers	18 (21)
Type of abuse	
Physical	59 (70)
Neglect	29 (35)
Sexual	28 (33)
Penetrative sexual assault among those sexually abused^a^	11 (39)
Psychological	67 (80)
Number of abuse types experienced	
1 type	23 (27)
2 types	34 (40)
≥ 3 types	27 (32)
Outcomes	
Hospitalization	48 (57)
Outpatient visits	36 (43)

Data are presented as median [interquartile range (IQR)] or unweighted number (percentage) of children. ^a^Percentages for indented items under “Sexual abuse” are calculated among children with sexual abuse (*n* = 28), not the total sample (*n* = 84)

### Prevalence of abuse

Psychological abuse was the type of abuse most commonly reported, followed by physical abuse, sexual abuse, and neglect. Among the 28 children who reported being sexually abused, 11 experienced penetrative sexual assault, indicating a substantial proportion of severe sexual violence within this category (Table 1). In terms of cumulative burden, 23 (27%) children experienced one type of abuse, 34 (40%) experienced two types, and 27 (32%) experienced three or more types of abuse.

### Gender-based differences in abuse

Significant gender-based differences were observed for both physical and sexual abuse (Table 2). Girls were notably more likely to report being physically and sexually abused than boys. No significant gender-based differences were found for neglect or psychological abuse.

**Table 2:** Gender-specific prevalences of different types of abuse

Type of abuse	Gender	Present	Absent	Total	OR (95% CI)	*p* value
Physical	Girls	51	13	64		0.0015
	Boys	8	12	20		
Neglect	Girls	22	42	64		1
	Boys	7	13	20		
Sexual	Girls	26	38	64	6.16 (1.32–28.8)	0.0136
	Boys	2	18	20		
Psychological	Girls	52	12	64		0.537
	Boys	15	5	20		

Data are presented as unweighted number of children unless otherwise indicated. All comparisons were conducted using Fisher’s exact test

*OR* odds ratio, *CI* confidence interval

### NDs and types of abuse

Children with NDs were significantly less likely to report being sexually abused than those without NDs (Table 3). No statistically significant differences in the prevalence of physical abuse, neglect, or psychological abuse were observed.

**Table 3:** Incidence of child abuse among those with and without neurodevelopmental disorders

Type of abuse	Neurodevelopmental condition	Present	Absent	Total	OR (95% CI)	*p* value
Physical	ND	20	13	33		0.146
	Non-ND	39	12	51		
Neglect	ND	13	20	33		0.488
	Non-ND	16	35	51		
Sexual	ND	6	27	33	0.29 (0.10–0.83)	0.0198
	Non-ND	22	29	51		
Psychological	ND	23	10	33		0.0947
	Non-ND	44	7	51		

Data are presented as unweighted number of children unless otherwise indicated. All comparisons were conducted using Fisher’s exact test

*OR* odds ratio, *CI* confidence interval, *ND* neurodevelopmental disorder

### Hospitalization and clinical characteristics

Hospitalization was significantly associated with the number of types of abuse experienced (Table 4). Children who were hospitalized had a higher median number of types of abuse than those who were not hospitalized. Girls were significantly more likely to be hospitalized than boys. No significant associations were observed between hospitalization and age, presence of an ND, or history of psychiatric outpatient visits.

**Table 4:** Relationship between hospitalization and clinical characteristics

	Hospitalization (+)	Hospitalization (−)	*p* value
*Relationship between hospitalization and children’s age and number of times of abuse* ^a^			
Age (years)	14 [13–15]	13.5 [12–14]	0.249
Number of times of abuse	2 [2–3]	2 [1–2]	0.00778

	Hospitalization (+) (*n* = 48)	Hospitalization (−) (*n* = 36)	*p* Value
*Relationship between hospitalization and children’s gender*,* presence of neurodevelopmental disorders*,* and history of psychiatric visits*^b^			
Gender			
Girls	41	23	0.042
Boys	7	13	
Neurodevelopmental conditions			
ND	16	17	0.287
Non-ND	32	19	
History of psychiatric visits			
Present	23	11	0.168
Absent	25	25	

Data are presented as median [IQR] or as unweighted number of children unless otherwise indicated. Comparisons were conducted using the ^a^Wilcoxon rank-sum test and ^b^Pearson’s chi-square test

*IQR* interquartile range, *ND* neurodevelopmental disorder

### Sexual abuse and related factors

Sexual abuse was not significantly associated with age (Table 5). Children with a history of sexual abuse had significantly higher odds of being hospitalized and were more likely to report sexual violence by non-caregivers. Children with NDs were significantly less likely to report being sexually abused.

**Table 5:** Relationship between sexual abuse and related factors

	Sexual abuse (+)	Sexual abuse (−)	*p* value		
*Relationship between sexual abuse and children’s age* ^a^					
Age (years)	13 [13–14]	14 [12–15]	0.571		

	Sexual abuse (+) (*n* = 28)	Sexual abuse (−) (*n* = 56)	Total	OR (95% CI)	*p* value
*Relationship between sexual abuse and hospitalization and sexual violence by non-caregivers* ^b^					
Hospitalization					
Present	22	26	48	4.23 (1.49–12.00)	0.0101
Absent	6	30	36		
Sexual violence by non-caregivers					
Present	11	7	18	4.53 (1.51–13.60)	0.0111
Absent	17	49	66		

Data are presented as median [IQR] or as unweighted number of children unless otherwise indicated. Comparisons were conducted using the ^a^Wilcoxon rank-sum test and ^b^Pearson’s chi-square test

*OR* odds ratio, *CI* confidence interval, *IQR* interquartile range

Additional descriptive statistics stratified by gender and presence of neurodevelopmental disorders are provided in Table S1.

## Discussion

This study provides critical insights into the clinical characteristics of abused children referred by CGCs to a child psychiatry department in Japan. Psychological abuse was the most prevalent form of abuse reported, followed by physical abuse, neglect, and sexual abuse. Significant gender differences were identified, with girls being more likely to be both physically and sexually abused (OR for sexual abuse = 6.16, 95% CI 1.32–28.8). Notably, sexual abuse was less frequently reported by children with NDs than by those without (OR 0.29, 95% CI 0.10–0.83). Psychiatric hospitalization was associated with both the cumulative number of types of abuse and the presence of sexual abuse, particularly when perpetrated by non-caregivers. Children who had experienced sexual abuse were also more likely to be hospitalized (OR 4.23, 95% CI 1.49–12.00) and to report sexual violence by non-caregivers (OR 4.53, 95% CI 1.51–13.60).

Our findings are also consistent with previous empirical and theoretical research indicating that early exposure to multiple forms of maltreatment increases vulnerability to psychiatric disorders [3, 8]. Recent meta-analyses further corroborate these associations, particularly concerning CSA. For example, Boumpa et al. [5] established a strong link between CSA and post-traumatic stress disorder across diverse cultural and gender populations, whereas Alves et al. [4] identified increased risks of self-harm and suicidality among CSA survivors. The high prevalence of sexual abuse that we observed among girls is consistent with global meta-analyses reporting gender disparities in patterns of sexual victimization [2, 21].

Our observation that children with ND were less likely to report sexual abuse is somewhat unexpected in light of prior international research. Large-scale studies have shown that children with NDs, including autism spectrum disorder and attention deficit/hyperactivity disorder, are at elevated risk for coercive sexual victimization [22, 23]. Several factors may account for the discrepancy between our findings and previous studies. Children with ND may experience difficulties recognizing or verbalizing abuse due to communication impairments or atypical social cognition, contributing to potential underreporting. Institutional procedures, cultural norms surrounding disclosure, and variations in professional training may also influence reporting patterns. Methodological differences—including sampling frames, settings, and criteria for abuse assessment—may further explain divergent prevalence estimates. Importantly, many children in our sample were evaluated by board-certified child psychiatrists, which likely reduced diagnostic overshadowing. Although our data suggest a lower reported prevalence of sexual abuse among children with ND, this finding requires cautious interpretation and warrants further investigation using diverse methodological approaches. Additionally, prior international research has shown that boys may delay or avoid disclosing sexual abuse due to gender norms and stigma, which could also contribute to lower reporting rates.

The strong association observed between sexual abuse and psychiatric hospitalization is consistent with studies from Western contexts, which have identified childhood sexual trauma as a factor associated with acute psychiatric crises requiring inpatient care [10]. Our findings further showed that sexual violence by non-caregivers was more prevalent among children who had experienced sexual abuse. This underscores the importance of monitoring risks beyond the immediate family, echoing WHO guidelines emphasizing assessment of school environments, online interactions, and community settings [1]. Caregiver-perpetrated sexual abuse is often regarded as particularly severe within the broader framework of CSA, because it entails not only sexual victimization but also a collapse of the child’s primary caregiving and protective systems. Such relational betrayal and loss of safety may heighten psychological distress and contribute to acute psychiatric presentations requiring intensive assessment or inpatient stabilization. Although our dataset did not include systematic indicators of family functioning or trauma-related sexualized behaviors, these well-documented clinical mechanisms may help explain why caregiver-perpetrated sexual abuse showed a strong association with psychiatric hospitalization in our cohort. Although prior research has rarely examined direct associations between CSA and psychiatric hospitalization, the severity of trauma-related symptoms may increase the likelihood of mental health service use, including inpatient treatment.

The present findings yield important clinical and systemic implications. First, the high prevalence of psychological abuse supports the need for routine psychological screening, even when physical injury is absent. Second, the gender-specific patterns of victimization highlight the value of gender-sensitive assessment protocols and support systems. Third, the association between the cumulative number of abuse types and psychiatric hospitalization underscores the cumulative impact of trauma and the importance of early, coordinated multidisciplinary interventions.

Although the reported prevalence of sexual abuse among children with ND was low, this should not be interpreted as evidence of reduced actual risk. Rather, it highlights the need for assessment strategies adapted to the communicative and cognitive characteristics of this population. Interagency collaboration has been shown to be essential in addressing child maltreatment, particularly for vulnerable groups such as children with NDs. Prior studies have identified structural and attitudinal barriers—including stigma, inconsistent information sharing, and limited interdisciplinary understanding—that hinder collaboration between child protection and mental health services [14, 17, 18]. Strengthening joint training opportunities, shared protocols, and professional trust may help improve the integration of psychiatric care within multidisciplinary responses. These findings suggest the potential value of standardized triage procedures and routine ND-sensitive screening protocols within CGC–psychiatry collaboration frameworks. Enhancing systematic recognition of extra-familial sexual victimization may further strengthen coordinated responses and ensure timely access to specialized mental health evaluation.

Although the institutional framework of CGCs is unique to Japan, the issues highlighted by this study—such as the importance of early psychiatric involvement, gender- and ND-sensitive assessments, and the cumulative impact of abuse—are relevant across systems. These observations may inform service delivery models in countries seeking to strengthen coordination between child protection and mental health care.

Several limitations warrant consideration. First, the retrospective design relied on existing documentation, which may have been incomplete or subject to reporting bias. Second, this single-center sample limits generalizability, as referral criteria are not standardized across CGCs and may vary across institutions. Third, the cross-sectional design precludes causal inference, and the study did not include longitudinal follow-up. Fourth, sociocultural norms may influence both detection and reporting of abuse. In addition, information on self-harm or suicide attempts was recorded for some cases but was not systematically available across all participants and therefore could not be analyzed quantitatively. Similarly, information regarding sibling-perpetrated abuse or the specific location of the abuse (e.g., home, school, or community settings) was not consistently documented in CGC referral materials, which limited our ability to examine intrafamilial patterns or contextual factors surrounding the abuse.

Furthermore, multivariate analyses were not conducted due to the modest sample size and categorical nature of key variables. Although bivariate analyses yielded important preliminary insights, future studies should employ multivariate regression models to adjust for potential confounding. Given the exploratory nature of this study and the number of comparisons performed, the possibility of false positives cannot be ruled out. Some variability in psychiatric diagnoses may exist, although the involvement of board-certified child psychiatrists likely reduced misclassification.

Future research should incorporate multicenter, prospective designs to validate the present findings and examine long-term mental health trajectories. Multivariate methods may help clarify interactions among types of abuse, gender, neurodevelopmental status, and clinical outcomes. Qualitative research may also provide insights into the lived experiences of abused children. Finally, international comparative studies may help elucidate how cultural and systemic factors shape recognition, reporting, and clinical responses to child maltreatment.

## Conclusions

The findings of this study highlight the clinical significance of gender differences, sexual abuse characteristics, and cumulative exposure to multiple types of maltreatment among children referred by CGCs to a child psychiatry department in Japan. These results also underscore that children with neurodevelopmental disorders may face unique challenges in recognizing or communicating experiences of sexual victimization. Although the study is situated within Japan’s CGC-based referral system, the patterns observed—particularly the strong association between sexual abuse, non-caregiver–perpetrated violence, and psychiatric hospitalization—may hold relevance for comparable multidisciplinary settings internationally. Overall, the findings reinforce the need for careful clinical assessment and coordinated collaboration between child protection and mental health services to ensure timely identification and support for this vulnerable population.

## Supplementary Information

Below is the link to the electronic supplementary material.

Supplementary Material 1: Table S1. Stratified descriptive statistics by gender and presence of neurodevelopmental disorders.

Supplementary Material 2: Fig. S1. Patient flow from CGC referral to psychiatric assessment and inclusion/exclusion for analysis.

## Data Availability

The data that support the findings of this study are not publicly available due to ethical restrictions and institutional policy on potentially identifiable clinical information. Aggregate data or additional tabulations can be shared upon reasonable request to the corresponding author and with approval from the institutional review board.

## Abbreviations

CGCs: Child Guidance Centers
CI: Confidence interval
CSA: Childhood sexual abuse
IQR: Interquartile range
MHLW: Ministry of Health, Labour and Welfare
ND: Neurodevelopmental disorder
OR: Odds ratio
WHO: World Health Organization
