# Supplementary material for: Clinical profiles and hospitalization patterns among abused children referred by Child Guidance Centers in Japan
Source: Discov Ment Health. 2025 Dec 18;6(1):14. doi: 10.1007/s44192-025-00360-w (PMC12830523; doi:10.1007/s44192-025-00360-w)
Supplement: Supplementary file 1 — Supplementary Material 1: Table S1. Stratified descriptive statistics by gender and presence of neurodevelopmental disorders. [file 44192_2025_360_MOESM1_ESM.docx]

**Table S1. Stratified descriptive statistics by gender and presence of neurodevelopmental disorders**

| Variable | Total (n = 84) | Girls (n = 64) | Boys (n = 20) | ND present (n = 33) | ND absent (n = 51) | p Value |
| --- | --- | --- | --- | --- | --- | --- |
| Age (years), median [IQR] | 14 [12–15] | — | — | — | — | — |
| Type of abuse |  |  |  |  |  |  |
| Physical | 59 (70) | 51 (80) | 8 (40) | 20 (61) | 39 (76) | 0.0015 (Gender); 0.146 (ND) |
| Neglect | 29 (35) | 22 (34) | 7 (35) | 13 (39) | 16 (31) | 1.000 (Gender); 0.488 (ND) |
| Sexual | 28 (33) | 26 (41) | 2 (10) | 6 (18) | 22 (43) | 0.0136 (Gender); 0.0198 (ND) |
| Penetrative sexual assaultᵃ | 11 (39)ᵇ | — | — | — | — | — |
| Psychological | 67 (80) | 52 (81) | 15 (75) | 23 (70) | 44 (86) | 0.537 (Gender); 0.0947 (ND) |
| Sexual violence by non-caregivers | 18 (21) | — | — | — | — | — |
| Outcomes |  |  |  |  |  |  |
| Hospitalization | 48 (57) | 41 (64) | 7 (35) | 16 (48) | 32 (63) | 0.042 (Gender); 0.287 (ND) |
| Outpatient visits | 36 (43) | — | — | — | — | — |
| History of psychiatric visits | 34 (41) | — | — | — | — | 0.168 (Hosp) |
| Number of times of abuse, median [IQR] | — | — | — | — | — | 0.00778 (Hosp) |

Note: Data are presented as median [interquartile range (IQR)] or unweighted number (percentage) of children unless otherwise indicated. p values correspond to Fisher’s exact test or Wilcoxon rank-sum test as used in the main tables. ᵃPercentages for indented items under “Sexual abuse” are calculated among children with sexual abuse (n = 28), not the total sample (n = 84). ᵇExpressed as n (%) within the sexual abuse subgroup.
