# Supplementary figures and images for: Clinical profiles and hospitalization patterns among abused children referred by Child Guidance Centers in Japan
Source: Discov Ment Health. 2025 Dec 18;6(1):14. doi: 10.1007/s44192-025-00360-w (PMC12830523; doi:10.1007/s44192-025-00360-w)

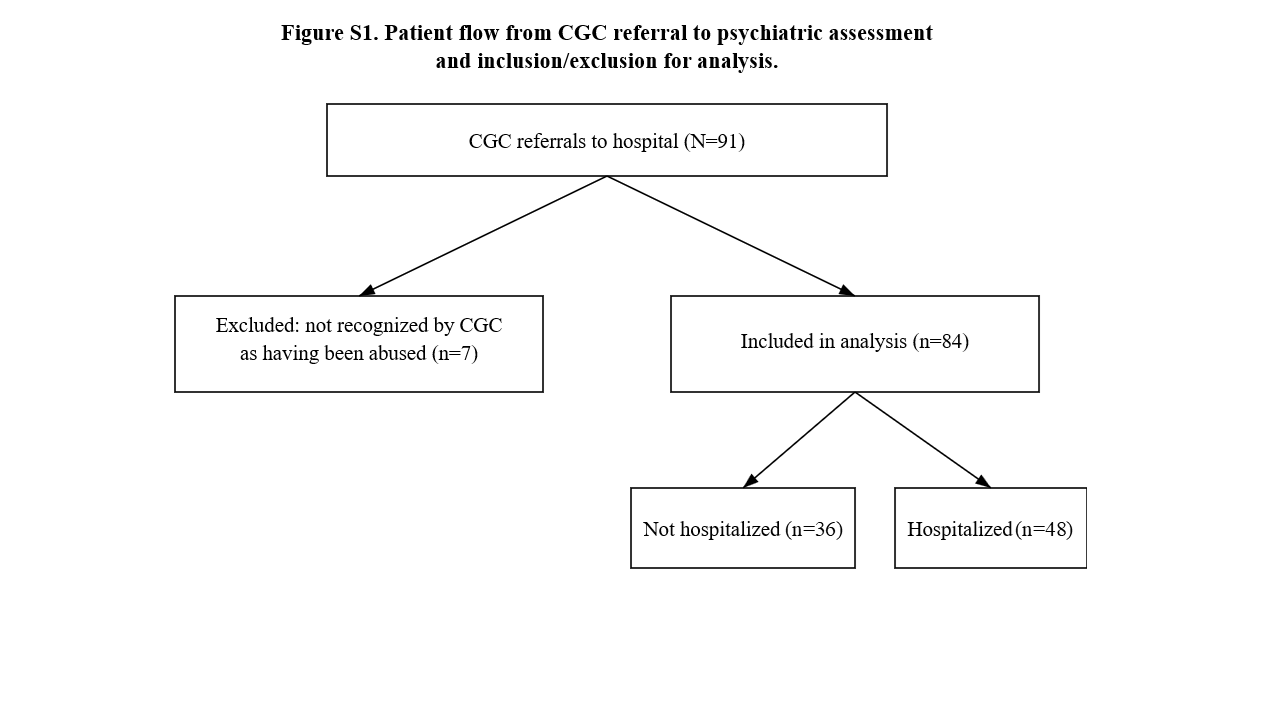

Supplement: Supplementary file 2 — Supplementary Material 2: Fig. S1. Patient flow from CGC referral to psychiatric assessment and inclusion/exclusion for analysis. [file 44192_2025_360_MOESM2_ESM.png]
